# Relationship between Target Time above Minimum Inhibitory Concentration Achievement Rate of Meropenem Using Monte Carlo Simulation and In-Hospital Survival in Patients with *Pseudomonas aeruginosa* Bacteremia

**DOI:** 10.3390/antibiotics13030219

**Published:** 2024-02-27

**Authors:** Hajime Nakashima, Motoyasu Miyazaki, Tsuneo Kuwamura, Kazutaka Oda, Yumi Haga, Osamu Imakyure

**Affiliations:** 1 Department of Pharmacy, Japan Community Health Care Organization Kyushu Hospital, Fukuoka 806-0034, Japan; 2 Department of Pharmacy, Fukuoka University Chikushi Hospital, Fukuoka 818-8502, Japan; motoyasu@fukuoka-u.ac.jp (M.M.); imakyure@fukuoka-u.ac.jp (O.I.); 3 Department of Pharmacy, Japan Community Health Care Organization Kurume General Hospital, Fukuoka 830-0013, Japan; tkuwamura1972@gmail.com; 4 Department of Pharmacy, Kumamoto University Hospital, Kumamoto 860-8556, Japan; kazutakaoda@kuh.kumamoto-u.ac.jp; 5 Department of Clinical Laboratory, Japan Community Health Care Organization Kyushu Hospital, Fukuoka 806-0034, Japan; haga-yumi@kyusyu.jcho.go.jp

**Keywords:** meropenem, *Pseudomonas aeruginosa*, bacteremia, Monte Carlo simulation

## Abstract

*Pseudomonas aeruginosa* bacteremia is associated with a high mortality rate, and meropenem (MEPM) is commonly used to treat it. However, the relationship between the time above the minimum inhibitory concentration (*f*T_>MIC_) of MEPM and its therapeutic efficacy in *P. aeruginosa* bacteremia has not been explored. This study aimed to investigate this relationship by defining the target % *f*T_>MIC_ of MEPM as 75%. The retrospective study spanned 14 years and included hospitalized patients treated with MEPM for *P. aeruginosa* bacteremia. Monte Carlo simulation was used to calculate the probability of target attainment (PTA) for each patient, and the threshold for a PTA of 75% *f*T_>MIC_ associated with in-hospital survival was determined using receiver operating characteristic (ROC) curves. The ROC curve-derived PTA associated with improved in-hospital survival was 65.0%, a significant finding in multivariate logistic regression analysis adjusted for patient background factors (odds ratio: 20.49, 95% confidence interval: 3.02–245.23, *p* = 0.005). This result suggests a dosing regimen that achieves a PTA of at least 65% when the target *f*T_>MIC_ of MEPM for treating *P. aeruginosa* bacteremia is defined as 75%.

## 1. Introduction

Gram-negative bacteria have been reported in over 40% of all bloodstream infections [1], with *Pseudomonas aeruginosa* accounting for 3–7% of all bacteremia and 23–26% of Gram-negative bacteria-induced bacteremia [2,3,4]. Furthermore, mortality rates for *P. aeruginosa*-induced bacteremia range from 18% to 46% [2,4,5,6,7,8]. Risk factors for *P. aeruginosa* bacteremia, categorized as a glucose nonfermenting Gram-negative rod, include hematologic malignancies like leukemia, chronic kidney disease, organ transplantation, and other immunosuppressive conditions [9,10]. Common sources of bacteremia include pulmonary, central line-associated, urinary tract, pancreaticobiliary, and unknown origins [10,11,12,13,14]. While antimicrobial susceptibility is crucial for treating infections, *P. aeruginosa* is intrinsically resistant to some commonly used antibiotics and can develop resistance to multiple antibiotic classes [15,16,17,18]. Therefore, the limited availability of antibiotics for *P. aeruginosa* infections complicates treatment, emphasizing the importance of appropriate antimicrobial therapy for *P. aeruginosa* bacteremia [6,11].

Meropenem (MEPM), a carbapenem, possesses a broad antibacterial spectrum and finds widespread use in antimicrobial therapy for various bacterial infections, including *P. aeruginosa* bacteremia [19,20]. Similar to other β-lactams, MEPM demonstrates time-dependent bactericidal activity. Its microbial efficacy is assessed based on the duration of time in percentage between doses at which the unbound drug concentration remains above the minimum inhibitory concentration (MIC), i.e., the time above the MIC (% *f*T_>MIC_) [21,22,23]. Furthermore, achieving *f*T_>MIC_ of 20–30% and 40–50% for MEPM is necessary for its bacteriostatic and bactericidal activities, respectively [24,25]. Few studies have reported a connection between the pharmacokinetics (PK)/pharmacodynamics (PD) parameter and clinical outcomes [26,27]. In critically ill patients, a higher survival rate has been reported in those with 75–100% *f*T_>MIC_ for MEPM compared to those with 40% *f*T_>MIC_ [26]. Among patients with febrile neutropenia, a higher clinical response rate was observed in those with >75% *f*T_>MIC_ for MEPM than in those with 50–75% *f*T_>MIC_ [27]. These findings indicate that achieving >75% *f*T_>MIC_ for MEPM is essential for enhancing therapeutic efficacy in severe infections.

Although it is necessary to know the drug concentration profiles at the target site while calculating the detailed PK/PD parameters, MEPM concentration measurements are still at the spreading stage. Therefore, it is crucial to predict the PK/PD parameters of antimicrobials for each patient based on available information and design appropriate dosage regimens. Monte Carlo simulation, a stochastic method utilizing random numbers, serves as an evaluation technique. In antimicrobial treatment, the probability of target attainment (PTA) of PK/PD parameters for various dosage regimens, including dosage and infusion time, can be determined based on population PK parameters and susceptibility results of organisms [26,28,29,30,31]. If the target value for efficacy, akin to achieving 75% *f*T_>MIC_ in the treatment of *P. aeruginosa* bacteremia with MEPM, is considered, the Monte Carlo simulation’s target achievement rate for patients on MEPM becomes a crucial efficacy indicator.

In this study, we utilized the reported population PK parameters for MEPM to estimate individual PK, using patient information with *P. aeruginosa* bacteremia. We then calculated PTA for each patient, targeting a value of 75% *f*T_>MIC_. Furthermore, we explored the correlation between the calculated PTA threshold and the outcomes of patients with *P. aeruginosa* bacteremia.

## 2. Results

### 2.1. Patient Background

**Figure 1:**
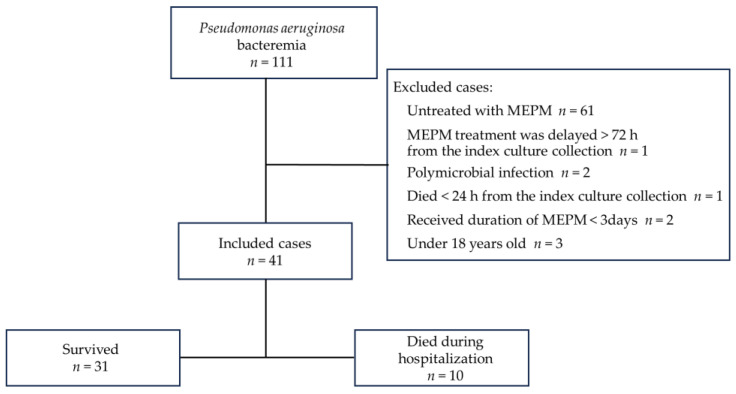
Study flow chart.

In total, 111 patients with *P. aeruginosa* bacteremia were screened for this retrospective study from January 2009 to December 2022, and 41 fulfilled the inclusion criteria, excluding 70 patients who met the exclusion criteria (Figure 1).

The patient background is described in Table 1. The median (interquartile range (IQR)) age of the patients was 77 (70–84), 70.7% were male, and the median (IQR) modified acute physiology and chronic health evaluation II (APACHE II) score at the time of collection of blood for the index culture was 20 (13–22). The median (IQR) creatinine clearance (CLcr) was 45 (20.9–75.5) mL/min, and 16 patients (39.0%) had acute kidney injury (AKI). Comorbidities included hypertension, chronic kidney disease (CKD), and hematologic malignancies in approximately 40% of the patients. Urinary tract infection (UTI) and unknown causes were the most common sources of bacteremia (34.1% [*n* = 14/41]).

The primary outcome, in-hospital survival, occurred in 31 (75.6%) of eligible patients. The duration of MEPM treatment did not differ significantly between survivors and non-survivors (median (IQR) of 8 (5.5–13) days and 10 (9–16) days, respectively; *p* = 0.171). Similarly, there was no significant distinction between the two groups regarding the concomitant use of other antimicrobials active against *P. aeruginosa* (12.9% [*n* = 4/31], 30% [*n* = 3/10]; *p* = 0.332). Conversely, patients who survived exhibited significantly lower modified APACHE II scores than patients who died (median (IQR) of 18 (12–22) and 22 (21–27), respectively; *p* = 0.005).

**Figure 2:**
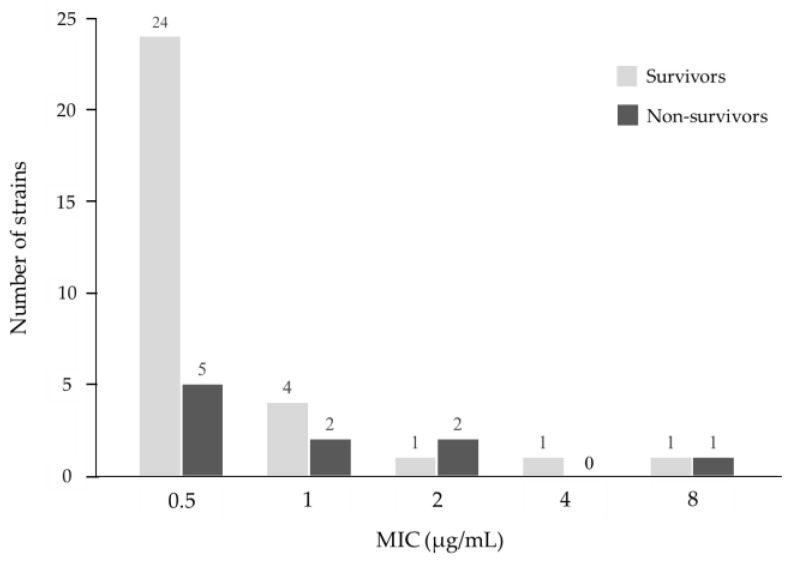
Minimum inhibitory concentration (MIC) distribution of meropenem for *P. aeruginosa* in this study.

The MIC distribution of MEPM for *P. aeruginosa* is described in Figure 2. In the isolated strains, 90.3% (*n* = 28/31) of the survivor strains and 70.0% (*n* = 7/10) of the non-survivor strains had MIC ≤ 1 μg/mL. The overall MIC_50_ and MIC_90_ values were 0.5 μg/mL and 2 μg/mL, respectively.

### 2.2. Probability of Target Attainment

**Figure 3:**
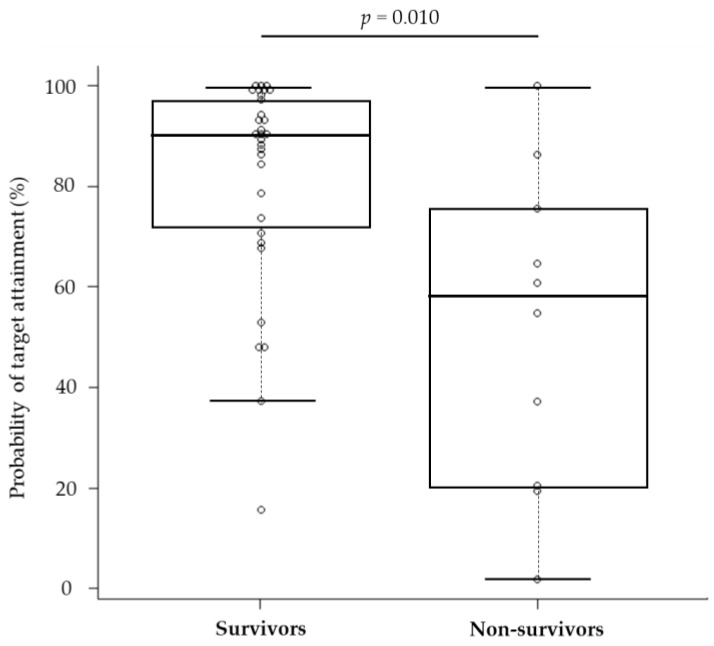
Comparison of the probability of target attainment between survivors and non-survivors. The complot shows the maximum value, 75% tile value, median value, 25% tile value, and minimum value in order from the top. The white circle symbol indicates the individual value.

**Figure 4:**
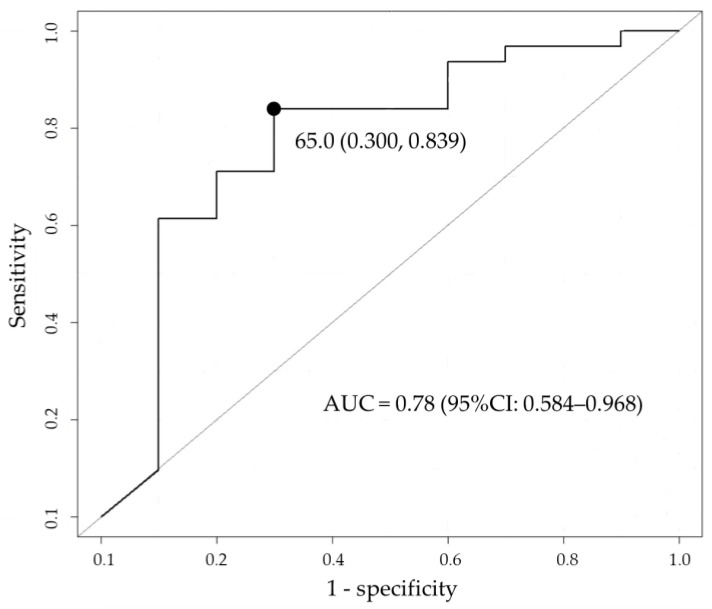
Cutoff value of probability of target attainment (PTA) that influences in-hospital survival based on receiver operating characteristic (ROC) analysis. The black circle indicates the optimal threshold. AUC, area under the curve; CI, confidence interval.

The median (IQR) PTA for 75% *f*T_>MIC_ was 90.2% (72.0–97.4) and 57.6% (24.6–73.1) for survivors and non-survivors, respectively, which were significantly different (Figure 3). The PTA threshold for 75% *f*T_>MIC_ to determine the likelihood of survival was identified by the receiver operating characteristic (ROC) curve (Figure 4). The area under the curve was 0.78 (95% confidence interval [CI]: 0.584–0.968), and the optimal threshold was 65.0%, with sensitivity and specificity of 83.9% and 70.0%, respectively. From the abovementioned data, achieving a PTA of >65%, the threshold for 75% *f*T_>MIC_ derived from the ROC curve was assessed as a candidate factor that may improve patient survival.

### 2.3. Investigating the Influence of the Probability of Target Attainment on In-Hospital Survival by Adjusting Patient Background Factors

The multivariate logistic regression analysis model included the modified APACHE II score, comorbid hematologic malignancies, and UTI as the cause of bacteremia, with *p* ≦ 0.15 in univariate analysis between survivors and non-survivors. The ROC curve-derived PTA threshold for 75% *f*T_>MIC_ was included as an independent variable. A PTA threshold of >65% was introduced into the final model as a categorical variable. The results indicated improved in-hospital survival for those with a PTA threshold of >65% (odds ratio [OR]: 20.49, 95% CI: 3.02–245.23, *p* = 0.005) and a modified APACHE II score (OR: 0.83, 95% CI: 0.69–0.96, *p* = 0.024) after controlling for clinical covariates (Table 2).

## 3. Discussion

The key finding of this study is that the ROC curve-derived threshold of PTA of >65%, with the target % *f*T_>MIC_ of MEPM defined as 75%, influences the improvement in in-hospital survival in *P. aeruginosa* bacteremia.

In total, 10 patients died in our study; all were receiving antimicrobial therapy since blood collection for the index culture till death, suggesting that all deaths were affected by infection. Among the 10 patients who died, 3 were in the intensive care unit (ICU) during the index blood culture collection. For the other patients, three out of seven were moved to the ICU a few days later for treatment, and four were treated in the regular ward because their condition was considered too futile to warrant ICU admission. Conversely, 2 out of the 27 survivors who were not initially admitted to the ICU were later transferred to the ICU. In 25 cases, the attending physicians decided to treat the patients in the regular ward due to their relatively stable vital signs.

In a study on the relationship between % *f*T_>MIC_ and the clinical efficacy of MEPM, Boonpeng et al. reported that, in the ICU, the number of patients with sepsis treated with MEPM achieving 40% *f*T_>MIC_, 75% *f*T_>MIC_, and 100% *f*T_>MIC_ was 92.9%, 71.4%, and 71.4%, respectively, in the survivor group and 83.3%, 66.7%, and 16.7%, respectively, in the non-survivor group [26]. Despite reporting more resistant organisms in the non-survivor group, these results suggest that an increase in % *f*T_>MIC_ of MEPM in critically ill patients may be associated with improved survival. The median (IQR) APACHE II score of the patient population in their study was 20 (14–23). In the present study, 17.1% of the patients were admitted to the ICU, but the median (IQR) modified APACHE II score of 20 (13–22) was similar in severity to that of the Boonpeng et al. group. Ariano et al. reported that, in febrile neutropenia patients with bacteremia, the mean % *f*T_>MIC_ of 42 patients in the clinical responders was 83%, whereas the mean % *f*T_>MIC_ of 18 patients in the non-responders was 59% (*p* = 0.04). In the same report, they also stated that a rate of % *f*T_>MIC_ for MEPM exceeding 75% was associated with an improved clinical response [27]. Although their report did not adjust for patient severity or comorbidities, the neutropenic status of their patients suggests susceptibility to infection. In the present study, 39.0% of the patients also had comorbid hematologic malignancies, suggesting that their background included patients with the same trends as those reported by Ariano et al. Hence, it was considered appropriate to set the target % *f*T_>MIC_ for MEPM at 75% in this study.

Sjövall et al. employed a Monte Carlo simulation to examine MEPM dosing in septic shock patients with potential augmented renal clearance [32]. They found that achieving a sufficient 100% *f*T_>MIC_ against *P. aeruginosa* requires either a prolonged infusion of 1 g every 8 h with a 3 h infusion time or a continuous infusion of 3 g/day over 24 h. Fukumoto et al. noted that in sepsis patients, with an MIC of MEPM set at 4 μg/mL, 1 g per dose (infusion time: 3–8 h), three times a day is necessary to achieve a 50% *f*T_>MIC_ in patients with CLcr >85 mL/min [33]. Roberts et al. used Monte Carlo simulation based on the PK parameters of MEPM in septic patients without renal dysfunction to compare the achievement rates of 50% *f*T_>MIC_ among continuous, prolonged, and intermittent bolus infusions [34]. Reportedly, for MIC > 4 μg/mL, continuous infusion had the highest rate of achieving the target, followed by prolonged infusion; however, intermittent bolus infusion revealed the lowest achievement rate. Based on these findings, patients with conserved renal function and predicted higher MEPM clearance and/or organisms with MIC > 4 μg/mL may benefit from prolonged infusion times or continuous infusion to increase % *f*T_>MIC_. However, no studies explored the correlation between % *f*T_>MIC_ and clinical efficacy. As the Clinical and Laboratory Standards Institute (CLSI) criteria set the breakpoint for MEPM against *P. aeruginosa* at 2 μg/mL [35], clinical assessment becomes crucial when the MIC surpasses this level in the clinical setting. In this study, the MIC_50_ and MIC_90_ of MEPM against *P. aeruginosa* were 0.5 μg/mL and 2 μg/mL, respectively, indicating overall high susceptibility to MEPM. Furthermore, participants in this study had overall impaired renal function, with a median (IQR) CLcr of 45 (20.9–75.5) mL/min, suggesting that these factors may have significantly impacted the elevation of the patients’ PTA in this study.

For treating infections, drug concentrations in the infection site are also an important factor affecting clinical efficacy, and there are several reports on blood concentrations of MEPM and its penetration into various organs [36,37,38,39,40]. In a study of patients with severe pneumonia, the concentration of MEPM in epithelial lining fluid (ELF) was reported to be much lower than that in plasma, with a penetration rate to ELF of approximately 30% of that in blood [36,37]. Concurrently, it has been reported that the usual dosage of MEPM (1 g–2 g per dose every 8 h) does not achieve 40–50% *f*T_>MIC_ in the ELF for pathogens with MIC ≤ 2 μg/mL. Conversely, MEPM is mainly eliminated from the kidney, with the urinary excretion rate of unchanged MEPM in healthy adults being approximately 65% [41,42]. Therefore, higher drug concentrations can be obtained in tissues with urinary tract infections compared with infections in other organs. Based on these findings, it is possible that differences in the penetration of MEPM into the infected organ that caused the bacteremia in this study also affected the outcome of the treatment. In fact, higher survival rates were observed in the group of patients in whom urinary tract infection was considered the cause of bacteremia.

This study has several limitations. First, it is a single-center, retrospective study, making it challenging to adjust for all confounding factors affecting clinical outcomes, compounded by the small number of included patients. The limited sample size may have impacted the estimation of the adjusted odds ratio for PTA > 65% on in-hospital survival, as indicated by large confidence intervals in some results. Second, the study did not measure the actual blood levels of MEPM in patients; thus, it is not possible to consider the effect of the changes in individual patients’ pathophysiology on the PK of MEPM. Critical illness, such as septic shock, may cause changes in vascular hyperpermeability and decreased peripheral vascular resistance with subsequent tissue edema, leading to an increase in capillary-to-cell diffusion distances [43]. Additionally, aggressive fluid resuscitation in patients with sepsis will lead to significant changes in the volume of distribution of antibiotics, potentially resulting in low trough serum and tissue concentrations. Therefore, the population’s PK parameters used in this study did not accurately reflect the PK parameters of MEPM in critically ill patients [44]. Next, critically ill patients are likely to have changes in the clearance of drugs excreted by the kidney due to the concomitant augmented renal clearance or AKI [44,45]. Therefore, it is difficult to exactly assess the clearance of MEPM—which changes daily—by estimating the Ccr level using a mathematical model. Herein, 16 patients (39.0%) had AKI complications, and of these, 5 were admitted to the ICU. Especially in this patient group, the PK of MEPM changed during the bacteremia treatment period, which may have affected the PTA results. Despite these constraints, this is the first report examining the relationship between the target % *f*T_>MIC_ achievement rate of MEPM and in-hospital survival in *P. aeruginosa* bacteremia. We believe our findings will contribute to the appropriate dosage design for MEPM. The study suggests that defining the target % *f*T_>MIC_ of MEPM as 75% and achieving PTA of >65% may enhance in-hospital survival in patients with *P. aeruginosa* bacteremia. As we have shown, PTA cannot be predicted using data on dose alone. Most of the *P. aeruginosa* bacteria isolated in this study were susceptible to MEPM with MIC ≦ 1 μg/mL, but investigating the dosing methods, such as continuous or prolonged dosing, may be required for more resistant pathogens in future studies. Furthermore, the population’s PK parameter applied for calculating PTA using the Monte Carlo simulation method are quite important, and for critical diseases such as sepsis, it may be necessary to use the population’s PK parameters constructed using PK data for individual diseases. We hope that the results obtained in the present study will serve as the foundation for further investigations.

## 4. Conclusions

When MEPM concentration monitoring is still in the spreading stage, defining a target % *f*T_>MIC_ and calculating PTA using an appropriate PK model based on patient information and pathogen’s MIC could enable the prediction of a dosing regimen that will result in a higher clinical efficacy. It is recommended to design MEPM dosing for patients with *P. aeruginosa* bacteremia to achieve a PTA of >65%, defined as a target 75% *f*T_>MIC_.

## 5. Materials and Methods

### 5.1. Study Population

This retrospective study included patients with positive blood cultures for *P. aeruginosa* admitted to Japan Community Health Care Organization Kyushu Hospital from January 2009 to December 2022. Patients < 18 years of age, who were untreated with MEPM, subjected to delayed treatment beyond 72 h from the index blood culture collection, treated with MEPM for less than 3 days, and those with polymicrobial blood culture findings were excluded. Patients who died within 24 h of blood culture collection were also excluded.

### 5.2. Clinical Background

Patient characteristics and clinical outcomes were retrospectively retrieved from the electronic medical records. Information on *P. aeruginosa*-positive blood cultures, including the MIC for MEPM, was obtained from the microbiology laboratory database. The extracted data encompassed age, sex, body weight, admission to ICU versus non-ICU, modified APACHE II score [46], serum creatinine (Scr), presence of AKI, infection source, duration of hospitalization until the collection of the index blood culture, duration of MEPM therapy, receipt of active combination therapy with MEPM, and comorbidities. CLcr was calculated using the Cockcroft–Gault equation [47]. The source of infection referred to the attending physician’s written diagnosis. Patient comorbidities were considered present if documented in the admission history and physical examination. In-hospital survival was investigated as the outcome.

### 5.3. Definition

The day of positive blood culture collection was considered the first day of onset of bacteremia. In the presence of AKI, we adopted class 1 of the Acute Kidney Injury Network classification [48], defined as an increase in Scr level of > 0.3 mg/dL or >50% in at least two consecutive measurements during MEPM treatment. Combination antimicrobial therapy was defined as the administration of any other antimicrobial agent active against *P. aeruginosa*, following the CLSI criteria, concurrently with MEPM within the same 24 h period. MEPM dose intensities were categorized as 1 g every 8 h, 1 g every 12 h, 500 mg every 8 h, 500 mg every 12 h, or 500 mg every 24 h. MEPM doses, prescribed per the attending physician’s decision, were administered intravenously over 30 min to 1 h using a venous catheter. In-hospital survival was defined as the successful completion of antimicrobial therapy for bacteremia during hospitalization.

### 5.4. PK/PD Simulation of Individual Patients

Population PK parameters based on the two-compartment model reported by Ikawa et al. were used to determine PK parameters for individual patients, with a plasma protein binding rate of 2.43% [49]. The population PK parameters used for the calculation are shown below. The residual variability was disregarded.

CL (L/h) = 0.0905 × CLcr + 2.03 (IIV: 41.1%)

Vc (L) = 0.199 × BW (IIV: 39.8%)

Q (L/h) = 4.02 (IIV: 32.8%)

Vp (L) = 4.55 (IIV: 29.9%)

PBR (%) = 2.43

CL, clearance; Vc, central volume of distribution; Q, intercompartmental clearance; Vp, peripheral volume of distribution; PBR, blood plasma protein binding ratio; CLcr, creatinine clearance (L/h); BW, body weight (kg); and IIV, interindividual variability.

The PTA for individual patients was calculated using Monte Carlo simulation (*n* = 10,000) with a target value of 75% *f*T_>MIC_, which was reported to be highly effective in critically ill and febrile neutropenia patients [26,27]. The PK/PD simulation software BMs-Pod version 8.06 was used for Monte Carlo simulation [50].

### 5.5. Statistical Analysis

The threshold of PTA for 75% *f*T_>MIC_ linked to improved survival was determined through a ROC curve. To assess the correlation between the PTA threshold for 75% *f*T_>MIC_ derived from the ROC curves and the survival outcome in patients with *P. aeruginosa* bacteremia, a multivariate logistic regression analysis was conducted after adjusting for significant patient background factors. For the selection of patient background factors in the comparison between survivors and non-survivors, the Mann–Whitney U test was employed for continuous variables, and Fisher’s exact probability test was used for categorical variables. Candidate variables with a univariate significance level of *p* ≤ 0.15 were assessed as the potential predictors of in-hospital survival, and the stepwise method was applied for variable imputation in the multivariate logistic regression analysis. Statistical analysis was carried out using R version 4.3.1 (https://cran.r-project.org/, accessed on 3 May 2023).

## Figures and Tables

**Table 1:** Characteristics of 41 patients with *P. aeruginosa* bacteremia.

	Value for the Following Groups:			
Characteristic	Total Cohort (*n* = 41)	Survivors (*n* = 31)	Non-Survivors (*n* = 10)	*p* Value
Age in years, median (IQR)	77 (70–84)	79 (69–86)	73 (70–78)	0.230
Male, *n* (%)	29 (70.7)	21 (67.7)	8 (80.0)	0.694
Wt (kg), median (IQR)	55.9 (47.5–63.3)	55.9 (47.6–63.3)	52.9 (48.1–61.7)	0.767
Modified APACHE II score on the day of culture, median (IQR)	20 (13–22)	18 (12–22)	22 (21–27)	0.005
Creatinine clearance on the day of culture (mL/min), median (IQR)	45 (20.9–75.5)	40.3 (18.8–62.4)	66.1 (32.1–80.0)	0.300
Patients with AKI, *n* (%)	16 (39.0)	12 (38.7)	4 (40)	1
ICU at culture, *n* (%)	7 (17.1)	4 (12.9)	3 (30)	0.332
Days to positive culture from admission, median (IQR)	14 (0–24)	14 (0.5–24.5)	16 (0–19.25)	0.854
Duration of meropenem therapy, median (IQR)	9 (6–14)	8 (5.5–13)	10 (9–16)	0.171
Received active combination therapy with meropenem, *n* (%)	7 (17.1)	4 (12.9)	3 (30)	0.332
Patients with the following medical history, *n* (%)				
Hypertension	18 (43.9)	14 (45.2)	4 (40)	1
Type 2 diabetes mellitus	13 (31.7)	9 (29)	4 (40)	0.698
Ischemic heart disease	7 (17.1)	6 (19.4)	1 (10)	0.660
Heart failure	7 (17.1)	7 (22.6)	0 (0)	0.164
Cerebrovascular disease	4 (9.8)	3 (9.7)	1 (10)	1
CKD	16 (39.0)	13 (41.9)	3 (30)	0.712
Solid tumors	10 (24.4)	8 (25.8)	2 (20)	0.622
Hematological malignancies	16 (39.0)	10 (32.3)	6 (60)	0.150
Source, *n* (%)				
Respiratory	7 (17.1)	5 (16.1)	2 (20)	1
Urinary	14 (34.1)	13 (41.9)	1 (10)	0.123
Intra-abdominal	5 (12.2)	4 (12.9)	1 (10)	1
Skin and wounds	1 (2.4)	0 (0)	1 (10)	0.244
Unknown	14 (34.1)	9 (29.0)	5 (50)	0.267

IQR, interquartile range; APACHE II, acute physiology and chronic health evaluation II; AKI, acute kidney injury; ICU, intensive care unit; and CKD, chronic kidney disease.

**Table 2:** Logistic regression model of in-hospital survival.

Parameter	Adjusted OR for In-Hospital Survival	95% CI	*p* Value
PTA > 65%	20.49	3.02–245.23	0.005
modified APACHE II score	0.83	0.69–0.96	0.024
UTI as a cause of bacteremia	6.05	0.57–169.07	0.186

Model χ^2^ test, *p* < 0.01, Hosmer–Lemeshow test, *p* = 0.605. OR, odds ratio; CI, confidence interval; PTA, probability of target attainment; APACHE II, acute physiology and chronic health evaluation II; and UTI, urinary tract infection.

## Data Availability

Data are contained within the article.
